# Using New and Innovative Technologies to Assess Clinical Stage in Early Intervention Youth Mental Health Services: Evaluation Study

**DOI:** 10.2196/jmir.9966

**Published:** 2018-09-10

**Authors:** Laura Ospina-Pinillos, Tracey Davenport, Frank Iorfino, Ashleigh Tickell, Shane Cross, Elizabeth M Scott, Ian B Hickie

**Affiliations:** 1 Brain and Mind Centre The University of Sydney Sydney Australia; 2 University of Notre Dame Australia Sydney Australia

**Keywords:** staging model, mental health, primary health care, telemedicine, symptom assessment health service reform

## Abstract

**Background:**

Globally there is increasing recognition that new strategies are required to reduce disability due to common mental health problems. As 75% of mental health and substance use disorders emerge during the teenage or early adulthood years, these strategies need to be readily accessible to young people. When considering how to provide such services at scale, new and innovative technologies show promise in augmenting traditional clinic-based services.

**Objective:**

The aim of this study was to test new and innovative technologies to assess clinical stage in early intervention youth mental health services using a prototypic online system known as the Mental Health eClinic (MHeC).

**Methods:**

The online assessment within the MHeC was compared directly against traditional clinician assessment within 2 Sydney-based youth-specific mental health services (*headspace* Camperdown and *headspace* Campbelltown). A total of 204 young people were recruited to the study. Eligible participants completed both face-to-face and online assessments, which were randomly allocated and counterbalanced at a 1-to-3 ratio. These assessments were (1) a traditional 45- to 60-minute *headspace* face-to-face assessment performed by a Youth Access Clinician and (2) an approximate 60-minute online assessment (including a self-report Web-based survey, immediate dashboard of results, and a video visit with a clinician). All assessments were completed within a 2-week timeframe from initial presentation.

**Results:**

Of the 72 participants who completed the study, 71% (51/72) were female and the mean age was 20.4 years (aged 16 to 25 years); 68% (49/72) of participants were recruited from *headspace* Camperdown and the remaining 32% (23/72) from *headspace* Campbelltown. Interrater agreement of participants’ stage, as determined after face-to-face assessment or online assessment, demonstrated fair agreement (kappa=.39, *P*<.001) with concordance in 68% of cases (49/72). Among the discordant cases, those who were allocated to a higher stage by online raters were more likely to report a past history of mental health disorders (*P*=.001), previous suicide planning (*P*=.002), and current cannabis misuse (*P*=.03) compared to those allocated to a lower stage.

**Conclusions:**

The MHeC presents a new and innovative method for determining key clinical service parameters. It has the potential to be adapted to varied settings in which young people are connected with traditional clinical services and assist in providing the right care at the right time.

## Introduction

Globally, there is increasing recognition that new strategies are required to reduce disability due to common mental health problems such as anxiety, depression, and comorbid substance misuse. As public awareness increases, the demand for mental health care far outstrips the capacity of health systems to provide access to quality care [1]. To achieve a meaningful reduction in population-level burden of disease, there is a need to provide both prevention and early intervention strategies at scale. As 75% of mental health and substance use disorders emerge during the teenage or early adulthood years [2], these strategies need to be readily accessible to young people. In most countries, however, young people are less likely to receive effective mental health care as a consequence of financial, attitudinal, and health system literacy factors [3,4].

For young people, there is typically still a prolonged delay between the onset of first symptoms and initial treatment contact [5]. By the time most young people present to health services, they already have significant functional impairment, are psychologically distressed, or have some degree of established comorbidity [6]. For these young people, the current psychiatric classification systems remain limited [7] and, as interventions are often guided by diagnosis, young people experiencing subthreshold symptomatology do not always receive appropriate care [8].

When considering how to provide such services at scale, eHealth (electonic health is a relatively recent health care practice that is supported by internet and/or other technologies) [9] and more recently mHealth (mobile health is the use of mobile and wireless technologies to enhance health) [10] and uHealth (ubiquitous health is information technology combined with medical technolgy to support health) [11] technologies show promise in augmenting traditional services and can be adapted potentially to all aspects of care [12]. A consistent theme is that such technologies can also address poor youth engagement with mental health services [13]. In these services, for example, innovations in the use of such technologies that allow the assessment process to be brought online could improve current wait times and provide a wider reach for young people who physically (or emotionally) struggle to access face-to-face clinical care [14].

While there are concerns about potential lack of accountability in the mHealth field and that online communications could miss nonverbal cues that can ultimately impact empathy and patient satisfaction [15], it is also evident that Web-based programs can facilitate disclosure [16] and such online interventions are as effective and even more efficient than traditional interventions in mental health [17,18]. Furthermore, evidence shows that telepsychiatry is as accurate as in-person psychiatry, and online users experience the same degree of satisfaction as face-to-face users [19].

Imported from general medicine, the concept and application of clinical staging to mental health disorders seeks to redefine traditional diagnostic systems by placing individuals on a spectrum from early identification of nonspecific or mixed forms of mental symptoms through to more discrete disorders and then recurrent and persistent forms of illness [20]. Transdiagnostic staging models have been employed in youth mental health settings and have demonstrated utility [8,21] (Textbox 1). To date, research among youth-focused primary mental health care services has shown that 33% to 41% of young people presenting to early intervention youth mental health services are assigned to stage 1a, 38% to 40% to stage 1b, 11% to 14% to stage 2 and 7% to 8% to stages 3 and 4 [8].

As the staging framework recognizes the continuum of illness progression, it also encourages more personalized and responsive care at each point of the spectrum [20]. This framework supports the promotion of self-help and encourages easier navigation for stepping up through the mental health system [22]. Stepped (or clinically staged) care aims to provide evidence-based, less intensive, low-risk, and low-cost interventions to the less severe cases while prioritizing more intensive or prolonged interventions for more complex cases [7,23-26].

The process for determining stages has been outlined previously by Cross and colleagues [8]. To briefly summarize the procedure, allocation of an individual to a particular stage is undertaken at regular multidisciplinary clinical consensus meetings involving senior mental health professionals (consultant psychiatrists or senior clinical psychologists) aided by objective symptom and functional measures (including paper and pencil questionnaires and surveys administered by tablets) and cross-referencing the staging framework set out by Hickie and colleagues [7]. Converting these methodologies to an online assessment (including a self-report Web-based survey, an immediate dashboard of results, and a video visit with a clinician) has the genuine potential to increase engagement with young people for many reasons. First, the internet is so widely used it is now the preferred mode of communication for youth [27,28].

**Clinical staging model for mental health disorders.**

- Stage 0 : no symptoms; person at risk of disorder
- Stage 1a: help-seeking; person with mild symptoms and mild functional impacts
- Stage 1b: attenuated syndrome; person with mixed or ambiguous symptoms and moderate to severe functional impacts
- Stage 2 : discrete disorders such as clear episodes of psychotic, manic, or severe depressive disorders
- Stage 3 : recurrent or persistent disorder
- Stage 4 : severe, persistent, and unremitting illness

Second, the assessment offers the possibility of immediate recommendations, support, and interventions anytime, anywhere, through a personalized dashboard of results (an easy-to-read clinical report with infographics) upon completion of the online assessment. Third, the assessment breaks down traditional geographical and socioeconomic barriers by increasing access to any care but specifically to more specialized assessment.

The aim of this study was to test new and innovative technologies to assess clinical stage in youth-specific mental health services using a prototypic online system known as the Mental Health eClinic (MHeC) [29]. Specifically, we tested how online assessments compared with traditional face-to-face assessments in a cohort of young people seeking mental health care. We report how online assessments perform in identifying key features such as stage allocation, lifetime trajectories, and recognition of comorbidities while also managing risk (suicidality) and responding to the more complex cases. The study compares the online assessment within the MHeC (including a self-report Web-based survey, an immediate dashboard of results, and video visit with a clinician) to standard face-to-face assessment as provided through 2 Sydney-based *headspace* services.

## Methods

### Participants

Participants were recruited from 2 youth-specific mental health services (*headspace* Camperdown and *headspace* Campbelltown) located in inner and outer metropolitan Sydney, Australia, respectively. *headspace* services are specialized, primary care early intervention mental health services for young people aged 12 to 25 years [30]. They provide services such as care coordination and support by allied health professionals; general medical services by general practitioners (primary care physicians); more specialized mental health services delivered by clinical psychologists and psychiatrists; and education, employment, and other social supports delivered by colocated specialist services. A key aspect of *headspace* is the direct connection and ease of access to secondary care specialists such as early psychosis services.

Within these *headspace* services, all young people who met inclusion criteria between the period of July 2015 to August 2016 were invited to participate in the study. Inclusion criteria included young people who (1) were aged 16 to 25 years, (2) were newly registered with *headspace*, (3) had regular access to the internet, and (4) had regular access to a webcam. Young people were reimbursed (voucher equivalent to Aus $20 [US $15]) for their participation.

### Ethics

The University of Sydney’s Human Research Ethics Committee approved the study (protocol number 2014/689). All participants were provided with information about the study prior to participating and consenting. Parental consent was also obtained for participants aged 16 and 17 years.

### Procedure

In order to test the online assessment within the MHeC, all eligible participants were invited to complete both the online assessment and standard assessment in face-to-face services. Participants were randomly allocated and counterbalanced by a 1-to-3 ratio to either undertake the face-to-face assessment or online assessment first. Considering the online assessment was a new method of assessment, an unequal randomization was preferred in order to minimize the impact of learning effects [31]. A condition of the study was that both assessments had to be completed within a 2-week timeframe from the first evaluation (the maximun interval of time in which symptomatology would not considerably differ between assessments).

The face-to-face assessment included completion of the *headspace* National Minimum Dataset (a very brief, 5- to 7-minute demographic and service activity questionnaire; data from this tool was not analyzed in this study) as collected through a survey administered via a tablet (smart skip rules are not available) upon entry to the service and then a 45- to 60-minute face-to-face psychosocial assessment with a Youth Access Clinician that is an adaptation of the HEEADSSS (Home, Education and Employment, Eating, Activities, Drugs and Alcohol, Sexuality, Suicide and Depression, Safety) [32] semistructured interview for *headspace*. This interview collects narrative information by assessing the young person’s home and environment and then progressively moves through the domains of education and/or employment, activities, drugs and alcohol, relationships and sexuality, conduct difficulties and risk taking, anxiety, eating, depression and suicide, and psychosis and mania [33-34] (Textbox 2).

The online assessment was based on the staging model as developed and adapted for early intervention youth mental health services. This assessment included 3 components (Textbox 3): a self-report Web-based survey, an immediate dashboard of results, and a video visit with a clinician. The self-report Web-based survey of the MHeC was designed and developed by our clinical research team to collect both demographic and clinical data. It is specifically ordered to reflect a best practice clinical interview [35] and includes 10 modules. Participants complete general questions about their demographics and medical history followed by questions assessing their current physical and mental health status. Questions are guided by smart skip rules that enable the self-report Web-based survey to be personally tailored to each young person (eg, if screening questions are positive, a more in-depth assessment will be triggered) and takes the minimum time to complete for each individual based on how they respond. Sensitive items (eg, suicidality and self-harm behaviors) are only asked once trust in the system has been generated and the young person is familiarized with the module’s topic and type of questions (ie, when rapport has been established).

**Face-to-face assessment.**

- *headspace* psychosocial assessment
  - Domain 1: home and environment
  - Domain 2: education and/or employment
  - Domain 3: activities
  - Domain 4: drugs and alcohol
  - Domain 5: relationships and sexuality
  - Domain 6: conduct difficulties and risk-taking
  - Domain 7: anxiety
  - Domain 8: eating
  - Domain 9: depression and suicide
  - Domain 10: psychosis and mania
- Face-to-face consultation with a clinician

**Online assessment.**

- Self-report Web-based survey
  - Module 1: collects demographic information
  - Module 2: assesses medical history
  - Module 3: screens for prevalent mental health conditions [36]
  - Module 4:
    - Screens for hypomanic symptoms (items derived from the Altman Self-Rating Mania Scale [37])
    - Screens for psychotic symptoms (items derived from the Community Assessment of Psychotic Experiences Positive Symptoms Scale [38])
    - Measures psychological distress with the 10-item Kessler Psychological Distress Scale [39]
    - Measures somatic distress with the Somatic and Psychological Health Report [40]
  - Module 5: Assesses self-harm behaviors and suicidality using the Suicidal Ideation Attributes Scale [41]
  - Module 6: Assesses tobacco, alcohol and substance use—items derived from the Alcohol Use Disorders Identification Test [42], the Alcohol, Smoking and Substance Involvement Screening Test [43], the Drinking Motives Measure [44], the Fagerstrom Nicotine Dependence Test [45], and select items from the National Household Drug Survey [46]
  - Module 7: Measures physical activity using the International Physical Activity Questionnaire [47]
  - Module 8: Assesses sleep behaviors using 4 sleep-related items from the Quick Inventory of Depressive Symptomatology [48]
  - Module 9: Assesses eating behaviors with items derived from the Eating Disorder Examination [49]
  - Module 10: Measures social connectedness—items derived from the Perceived Social Support/Conflict Measure [50] plus 5 items measuring relationship with peers [51]
- Immediate dashboard of results
- Video visit with a clinician

The video visit of the online assessment included a brief, semistructured interview (Multimedia Appendix 1) with clinicians (LOP, a psychiatrist and mental health researcher, and AT, a research psychologist) trained in the application of clinical staging [7] for young people presenting to *headspace* service. Importantly, the video visit was guided by the automatically generated results of the self-report Web-based survey as shown in the dashboard of results.

At the conclusion of each face-to-face session and video visit, all clinicians determined stage. These results were then collapsed into 2 groups: stage 1a or stage 1b and above (stage 1b+). Participants in stage 1a were help-seeking with mild symptoms and mild functional impairment while those in stage 1b+ were experiencing more severe symptoms and functional impairment. This key differentiation is predictive of clinical course and can be used to allocate service resources preferentially to those in greater need. Clinicians also completed the Social and Occupational Functioning Assessment Scale (SOFAS), which measures an individual’s functional status not directly related to the severity of their psychological symptoms [52], and the Clinical Global Impression Scale-Severity (CGI-S), a 7-point illness severity scale subjective to clinician’s past experience with other individuals with the same illness [53].

### Interrater Agreement Between the Online Clinicians

In order to validate online assessment and staging classification, 2 trained clinicians (LOP and AT) were present during all video visits until such time their interrater agreement was considered reliable; that is, LOP (rater A) and AT (rater B) conducted alternating video visits while the other was present but not in view of the webcam (as per ethics approval and consent obtained from the young person). Raters A and B then determined stage independently, and once substantial concordance was sufficiently reached, LOP and AT conducted any remaining video visits with or without the other present.

### Statistical Analyses

All statistical analyses were performed using SPSS Statistics for Mac 22.0 (IBM Corp). Group differences in demographic, functional, and clinical variables were assessed using nonparametric Kruskal–Wallis test (*H*) or chi-square test (χ^2^) at a 95% level of confidence (when the expected count was less than 5, a Fisher exact test [FET] was employed). Medians were reported due to sample size. Post hoc analyses (Mann–Whitney test [*U*], χ^2^, or FET) were performed in variables that showed significant differences between groups, using Bonferroni correction and adjusted alphas dependent on number of groups (n=3). *P* values less than .01 considered to be significant.

Interrater analyses determined degree of agreement of staging results between face-to-face and online clinicians as well as the 2 individual online clinicians (ie, rater A vs rater B). Cohen kappa statistic [54] was calculated and followed the interpretation criteria of Viera et al [55]: kappa=.01 to .20, slight agreement; kappa=.21 to .40, fair agreement; kappa=.41 to .60, moderate agreement; kappa=.61 to .80, substantial agreement; and kappa=.81 to .99, almost perfect agreement. For the continuous variables (SOFAS and CGI-S), the intraclass correlation coefficient (ICC) was used to calculate agreement between offline and online clinicians [56], and interpretations were based on a 95% confidence interval where estimates less than .50 reflect poor agreement; ICC=.50 to .75, moderate agreement; ICC=.75 to .90, good agreement; and greater than .90, excellent agreement [57].

## Results

### Recruitment and Participation

A total of 204 young people were identified as eligible to participate in the study. Based on a 1-to-3 random allocation counterbalancing ratio, 54 participants were invited to undertake standard face-to-face assessment first and 150 participants were invited to undertake the online assessment first; 125 participants were from *headspace* Camperdown and 79 from *headspace* Campbelltown. All were aged 16 to 25 years, and 71.6% (146/204) were female.

As shown in Multimedia Appendix 2, a total of 24% (13/54) of participants allocated to receive standard face-to-face assessment first completed both assessments, 46% (25/54) completed the standard face-to-face clinical assessment only, 19% (10/54) failed to complete either assessment, and the remainder withdrew from the study or were lost to follow-up. Conversely, 39.3% (59/150) of participants allocated to complete the online assessment first completed both assessments, 5.3% (8/150) completed the online assessment only, 49.3% (74/150) failed to complete either assessment, and the remainder withdrew from the study or were lost to follow-up. Overall, 72 participants completed the entire study protocol of which 68% (49/72) were recruited from *headspace* Camperdown and the remainder (23/72, 32%) from *headspace* Campbelltown. The average time to completion of the online assessment was 60 minutes including approximately 45 minutes (median 51 minutes) for the self-report Web-based survey and approximately 12 minutes (median 15 minutes) for the video visit.

### Sample Characteristics

The mean age of all participants was 20.35 (SD 2.63, range 16 to 25) years, 71% (51/72) were female, and 51% (37/72) had completed or partially completed tertiary education. Participants reported moderate distress levels (10-item Kessler Psychological Distress Scale mean 28.93, SD 8.42, range 10 to 50) with almost three-quarters (53/72, 74%) of the sample currently experiencing anxious and/or depressive symptoms. Nearly one-third (21/72, 29%) of participants screened positive for hypomanic symptoms, and one-third (24/72, 33%) screened positive for psychotic-like symptoms.

Almost half (35/72, 49%) of participants reported self-harm. Using our digitally smart Suicidality Escalation Protocol [58], the online assessment was able to detect and triage young people at risk in real time. In total, 18% (13/72) of participants reported high suicidality (Suicidal Ideation Attributes Scale [SIDAS] score ≥21/50), of which more than half (7/13, 54%) were escalated by the online clinicians to one of the *headspace* services as they considered current wait times for face-to-face care too long.

### Interrater Agreement Between Online Clinicians

In order to validate the online assessment and staging classification, the trained clinicians were both present in 14 video visits until agreement was measured as substantial (kappa=.76, *P*=.003) with concordance at 93% (13/14). All subsequent video visits were assessed by the raters according to their availability. The online interrater agreement was determined for 59% (48/82) of participants who completed the online assessment (self-report Web-based survey, immediate dashboard of results, and video visit with a clinician). As shown in Table 1, participants were entered into a 2-by-2 comparison of stage assigned (stage 1a vs stage 1b+) and type of online rater who assigned that stage: online rater A and online rater B. Overall agreement between online raters was measured as substantial (kappa=.77, *P*<.001) with concordance at 90% (43/48) upon completion of all online assessments; 82% (14/17) were classified by both online raters as stage 1a and 94% (29/31) as stage 1b+.

**Table 1:** Interrater agreement between online rater A and online rater B by assignment of clinical stage.

	Online rater A	
Online rater B	Stage 1a (n=16), n (%)	Stage 1b+ (n=32), n (%)
Stage 1a (n=17), n (%)	14 (29)	3 (6)
Stage 1b+ (n=31), n (%)	2 (4)	29 (61)

**Table 2:** Interrater agreement between face-to-face and online clinicians by allocation to clinical stage.

	Online assessment	
Face-to-face clinical assessment	Stage 1a (n=27), n (%)	Stage 1b+ (n=45), n (%)
Stage 1a (n=42), n (%)	23 (32)	19 (26)
Stage 1b+ (n=30), n (%)	4 (6)	26 (36)

### Face-to-Face Versus Online interrater Agreement

To calculate interrater agreement for assigning stage, participants were entered into a 2-by-2 comparison of stage assigned (stage 1a vs stage 1b+) and type of clinician who assigned that stage: face-to-face clinician versus online clinician (Table 2). Interrater agreement of stage between face-to-face and online clinicians demonstrated fair agreement (kappa=.39, *P*<.001), with concordance in 68% (49/72) of participants; here, clinicians identified 55% (23/42) stage 1a (agree) (staged as stage 1a by online and face-to-face clinicians) and 87% (26/30) stage 1b+ (agree) (staged as stage 1b by online and face-to-face clinicians). Of note, 1 participant was assigned stage 2 following face-to-face clinical care, and 3 participants were assigned stage 2 following the online assessment. In this study, no participants were assigned to the more severe stages (ie, stages 3 or 4). There was moderate interrater reliability in the SOFAS score between face-to-face and online clinicians (ICC=.73) and poor interrater reliability in the CGI-S allocation (ICC=.49) for all participants.

### Comparison of Self-Reported Measures Where There Was a Disagreement Between Face-to-Face Clinical Assessment and Online Assessment

Table 3 shows the main self-reported clinical characteristics across the 3 groups: stage 1a (agree); stage 1b+ (agree); and stage 1b+ (disagree). Stage 1b+ (disagree) refers to participants who were staged 1b+ by online clinicians but assessed as stage 1a by face-to-face clinicians.

#### Stage 1a (Agree) Versus Stage 1b+ (Disagree)

Comparing stage 1b+ (disagree) with those participants determined as stage 1a (agree) by both clinician types, post hoc analyses showed that almost all young people in stage 1b+ (disagree) reported a previous history of mental health problems (χ^2^_1_=10.71, *P*.001), and more than a third (7/19, 37%) reported they had a history of developing a suicide plan (*P*=.002; FET). With regard to current symptomatology, there were no significant differences in psychological distress or suicidal ideation. However, weekly cannabis use was higher in stage 1b+ (disagree) (*P*=.03; FET). Although both groups’ SOFAS (Table 4) scores were located within the same range (71 to 80: no more than a slight impairment in social, occupational, or school functioning), participants allocated to stage 1b+ (disagree) were consistently scored with lower levels of functioning (*U*=117, *z*=–2.58, *P*=.01) compared to those in stage 1a (agree).

There was also a major discrepancy between the face-to-face and the online clinicians in categorizing the symptom severity for the participants allocated to stage 1b+ (disagree) group; face-to-face clinicians considered this group as normal (not at all ill) whereas online clinicians assigned a mildly ill classification. Among the online observations, the symptomatology of this group was considered to be significantly more pronounced compared to the stage 1a (agree) participants (CGI-S median rating of borderline ill; *U*=68, *z*=–4.08, *P*<.001).

#### Stage 1b+ (Agree) Versus Stage 1b+ (Disagree)

When comparing stage 1b+ (disagree) with those in stage 1b+ (agree), post hoc analysis showed that participants assessed as stage 1b+ (agree) had significantly higher levels of suicidal ideation on the SIDAS (*U*=133.50, *z*=–2.64, *P=*.008) and lifetime self-harm behavior (χ^2^_1_=7.35, *P=*.007). According to online clinicians (Table 4), young people allocated to stage 1b+ (agree) had lower functioning levels on the SOFAS when compared with the stage 1b+ (disagree) group (*U*=121, *z*=–2.93, *P=*.003). However, the stage 1b+ (agree) group was classified by both assessment modes as more unwell on the CGI-S when compared with the stage 1b+ (disagree) group (face-to-face clinical care, *U*=55.5, *z*=–4.54, *P<*.001; online assessment, *U*=149, *z*=–2.48, *P=*.01). Previous mental health history, distress levels, alcohol and/or other substance use disorders, or comorbidities did not differ between these groups.

Post hoc analysis with stage 1a (disagree) (stage 1a by online clinicians but assessed as stage 1b+ by face-to-face clinicians) participants was not conducted due to insufficient cell size.

**Table 3:** Median scores and significance test results for self-reported variables among groups.

Characteristics			Stage 1a (agree)^a^ (n=23), n (%)	Stage 1b+ (agree)^b^ (n=26), n (%)	Stage 1b+ (disagree)^c^ (n=19), n (%)	Significance test *H*^d^ or FET^e^ (*P*)	Post hoc *P* values	
							a vs c	b vs c
**Demographics**								
	Female, n (%)		15 (65)	18 (69)	14 (74)	1.70^e^ (.72)	—^f^	—
	Age in years, median (IQR)^g^		20.00 (4)	20.50 (4)	21.00 (4)	0.78^d^ (.86)	—	—
	**Education**					1.58^e^ (.71)		
		Secondary, n (%)	12 (52)	14 (54)	8 (42)	—	—	—
		Tertiary, n (%)	11 (48)	12 (46)	11 (58)	—	—	—
**Clinical characteristics**								
	K-10^h^, median (IQR)		25 (13)	32.0 (9)	28.0 (13)	5.51^d^ (.14)	—	—
	Depression/anxiety (current), n (%)		16 (70)	22 (85)	14 (74)	6.03^e^ (.09)	—	—
	Hypomanic-like issue (current), n (%)		5 (22)	10 (38)	6 (32)	2.91^e^ (.38)	—	—
	Psychotic-like issue (current), n (%)		5 (22)	12 (46)	7 (37)	4.92^e^ (.15)	—	—
	Mental health history, n (%)		11 (48)	20 (77)	18 (95)	11.83^e^ (.005)	.001	.21
	Lifetime self-harm, n (%)		7 (30)	20 (77)	7 (37)	13.28^e^ (.003)	.67	.007
**Suicidality**								
	SIDAS^i^, median (IQR)		1 (4)	9.5 (24)	1 (5)	12.59^d^ (.006)	.71	.008
	Suicide planning history, n (%)		0 (0)	12 (46)	7 (37)	17.75^e^ (<.001)	.002	.53
	Suicide attempt history, n (%)		0 (0)	6 (23)	1 (5)	6.98^e^ (.04)	.45	.21
**Alcohol and/or other substance misuse**								
	Lifetime substance misuse, n (%)		17 (74)	18 (69)	14 (74)	0.36^e^ (.98)	—	—
	Cannabis weekly, n (%)		1 (4)	8 (31)	6 (32)	7.60^e^ (.04)	.03	.95
	Substances to cope with emotions, n (%)		6 (26)	18 (69)	8 (42)	10.85^e^ (.009)	.24	.07

^a^Stage 1a by online and face-to-face clinicians.

^b^Stage 1b+ by online and face-to-face clinicians.

^c^Stage 1b+ by online clinicians but assessed as Stage 1a by face-to-face clinicians.

^d^Kruskal–Wallis test, 2-tailed.

^e^FET: Fisher exact test, 2-tailed.

^f^Not applicable.

^g^IQR: Interquartile range.

^h^K-10: 10-item Kessler Psychological Distress Scale.

^i^SIDAS: Suicidal Ideation Attributes Scale.

**Table 4:** Median scores and significance test results for clinician-reported variables among groups.

Tests		Stage 1a (agree)^a^ (n=23), n (%)		Stage 1b+ (agree)^b^ (n=26), n (%)		Stage 1b+ (disagree)^c^ (n=19), n (%)		Significance test *H*^d^ (*P*)		Post hoc *P* values			
										a vs c		b vs c	
**CGI-S^e^**													
	Face-to-face, median (IQR)^f^		2.0 (1)		3.0 (2)		1.0 (1)		37.04 (<.001)		.83		<.001
	Online, median (IQR)		2.0 (1)		4.0 (1)		3.0 (1)		35.29 (<.001)		<.001		.01
**SOFAS^g^**													
	Face-to-face, median (IQR)		75.0 (9)		69.0 (15)		75.0 (5)		12.17 (.007)		.10		.08
	Online, median (IQR)		75.0 (9)		60.0 (10)		71.0 (10)		25.33 (<.001)		.01		.003

^a^Stage 1a by online and face-to-face clinicians.

^b^Stage 1b+ by online and face-to-face clinicians.

^c^Stage 1b+ by online clinicians but assessed as stage 1a by face-to-face clinicians.

^d^Kruskal–Wallis test, 2-tailed.

^e^CGI-S: Clinical Global Impression Scale–Severity.

^f^IQR: Interquartile range.

^g^SOFAS: Social and Occupational Functioning Assessment Scale.

## Discussion

### Principal Findings

The MHeC presents a new and innovative method for determining key clinical service parameters. While there was fair agreement between the staging classifications after both online and face-to-face assessment in the majority of cases (68%, kappa=.39), an important area of difference did emerge. During face-to-face assessments, clinicians tended to rate stage more conservatively compared to clinicians acting with the assistance of the MHeC.

Among the discordant cases, in 26% of cases face-to-face assessment appeared to place less emphasis on lifetime history of mental health problems. By contrast, the online assessment placed greater focus on past history of mental health problems *(P*=*.* 001), as well as any previous suicide planning (*P*=.002) and current comorbidity with cannabis misuse (*P*=.03) as indicators of progression of disease. It appears the online assessment process was a more efficient way of detecting lifetime severity by holistically evaluating these young participants’ current and previous mental health status.

There are a range of possible explanations for this important difference between the face-to-face and online assessments, including (1) face-to-face assessment places greater emphasis on current symptomatology, (2) online clinicians made specific use of more extensive data collection about past as well as current symptomatology that was collected prior to the video visit (and as a consequence, their clinical assessment used all available data relevant to assign stage), and/or (3) face-to-face clinicians may be more influenced by the consequences of their clinical assessment for allocating service resources—that is, higher stage ratings are reserved preferentially for those who are perceived to be in need of more intensive or prolonged care.

Assessing the mental health of young people and their need for immediate or ongoing health care is a real challenge for clinicians and youth mental health services. Specifically, this includes being able to distinguish normative emotional development and brief stress-related responses from emerging mental disorders [59,60] as well as obtaining accurate information from young people who may be apprehensive or hostile toward their clinician. Further, building rapport can take longer in this population [61], and clinician training is often based on the recognition of symptomatology leading to specific diagnostic (eg, *Diagnostic and Statistical Manual of Mental Disorders, 5th Edition*, or *International Classification of Diseases and Related Health Problems, 10th Revision*) constructs [62]. However, such categories do not accurately represent the most common admixtures of symptoms in young people presenting for mental health care. Clinicians working with the staging model are using a framework that proposes that once a person has reached a defined stage, it is not possible to return to an earlier stage, and as mental disorders are typically cyclical, complexity lies in understanding the variability of presentations over time. Therefore, clinicians would greatly benefit from accurate methods of collecting relevant staging information. The *headspace* psychosocial assessment used in practice predominantly focuses on the current symptomatology of young people and, as a result, misses relevant lifetime information that is crucial for staging.

By entering information online, young people can complete a self-report Web-based survey in their own time whenever and wherever they prefer. This provides greater choice at the forefront of mental health care by directly and immediately responding to young people’s needs [29]. For clinicians, this provides reliable information about the individual (current and lifetime) prior to a face-to-face assessment that can be used for staging, enabling clinicians to move away from traditional evaluations to more detailed data-driven assessments. This could translate into a more efficient way of assessment and improve the 1-on-1 time (face-to-face or video visit), enabling clinicians to expand and refine the information collected and deliver interventions that match a young person’s unique needs. Over time this online assessment process could be augmented by continuous data tracking and more detailed online assessments to help clinicians recognize patterns of symptoms in the data. Additionally, future systems could develop more complex algorithms through big data analyses and machine learning processes that can better inform young people, clinicians, and services.

As community-based and outpatient mental health care is limited, all services struggle with high demand pressures [63]. Consequently, users face long waiting lists that may have an adverse impact on their engagement with the service and ultimately increase the risk of hospitalizations, functional deterioration, self-harm, or suicidal behavior [64]. Additionally, clinicians and services face substantial demands to reduce waiting times while providing appropriate clinical care. Typically, service systems respond by prioritizing assessment, limiting the number of intervention sessions available and giving priority to more urgent cases [65]. We suggest that the difference in staging by face-to-face clinicians might also be contributed to by their practical awareness of such service constraints.

A systematic clinician bias toward underrating young people to stage 1a could have deleterious effects on service users. Our previous research has shown that 15% of people in stage 1b transition to stage 2 within 1 year [7], people in stage 1a receive different, shorter, and less intensive treatment compared with those in stage 1b [21], and young people in stage 1b tend to remain impaired and distressed over time [21]. The presence of past mental health and suicidal thoughts and/or behaviors indicates that this group of young people require a more personalized treatment that not only covers their current needs but responds appropriately to the higher stage they have reached over the course of their illness. An online assessment like the one proposed in the MHeC could assist to immediately identify young people who might benefit from seeing a more experienced clinician as soon as they enter a service for care. Consequently, such online assessment has the potential of transforming youth mental health services as it streamlines internal processes such as triage and evaluation, increases clinician capacity by providing immediate results, and matches the right clinician and intervention to the young person’s needs, thus ensuring the right care is provided at the right time.

Finally, one of the most obvious advantages of the online assessment addresses geographical barriers. In this trial, 10% (8/82) of the video visits were completed with 1 of our clinicians online while she was overseas (LOP traveled overseas due to work commitments) using secure videoconference software. This positions online assessment as an efficient solution connecting young people not only with care but with the right clinician regardless of their location, potentially saving time and money for young people, clinicians, and services.

### Limitations

One limitation of this study is the sample size because the face-to-face arm suffered from greater participant attrition. It is possible that participants who had already completed the face-to-face assessment felt that completing a second assessment online was an unnecessary use of time. Additionally, this study required people to complete all of the 4 main components (tablet questionnaire, face-to-face interview, Web-based survey, and video visit) within 2 weeks of the first interview, and the majority of attrition in both study arms was accounted for by this stringent protocol. Although the unequal randomization (1-to-3) favored the analysis with the reduction of the impacts of the learning curve, it compromised the power of the study. Future research is needed with a 1-to-1 randomization, increasing the power of the comparison.

Our study revealed poor interrater reliability on CGI-S allocation between face-to-face and online clinicians. There are 2 possible explanations for this disagreement. Face-to-face clinicians do not use the CGI-S in their daily practice and therefore are less familiar with its application, while online clinicians had used this tool in other research studies and were consequently more familiar with its application. Additionally, due to the CGI-S’s instruction (“Considering your total clinical experience with this particular population, how mentally ill is the patient at this time?”), it has been acknowledged that clinician experience could explain the variability in the CGI-S scoring [66]. It is important to note that our study used varying levels of clinicians (eg, psychiatrists vs less experienced Youth Access Clinicians) that could also act as confounding factors when scoring. Furthermore, this study reveals a difference between face-to-face and online clinicians, despite all clinicians having been trained in using the clinical staging framework as set out by Hickie and colleagues [7]. This suggests a need for an ongoing education and training program.

Future research is needed to evaluate the engagement, efficacy, and effectiveness of MHeC’s online assessment within real-world service environments. It would also include formal validation of the online assessment against gold standard assessment and testing the effectiveness of any education and training program that might be developed to supplement these new and innovative technological solutions for the delivery of better mental health care.

### Conclusions

This study highlights the use of new and innovative technologies to assess clinical stage in early intervention youth mental health services through an online MHeC. It promotes systematic assessment of lifetime severity and complexity of clinical presentations while concurrently addressing risk assessment in a shorter period of time. The MHeC has the potential to be adapted to varied settings in which young people are connecting with traditional clinical services and assist in providing the right care at the right time.

## Appendix

Multimedia Appendix 1 Video visit semistructured interview.

Multimedia Appendix 2 Consolidated Standards of Reporting Trials diagram indicating the flow of participants through the study.

## Abbreviations

CGI-S: Clinical Global Impression Scale-Severity
FET: Fisher exact test
H: Kruskal–Willis test
HEEADSSS: Home, Education and Employment, Eating, Activities, Drugs and Alcohol, Sexuality, Suicide and Depression, Safety
ICC: intraclass correlation coefficient
IQR: interquartile range
MHeC: Mental Health eClinic
SIDAS: Suicidal Ideation Attributes Scale
SOFAS: Social and Occupational Functioning Assessment Scale
U: Mann–Whitney test
