# Supplementary material for: Using New and Innovative Technologies to Assess Clinical Stage in Early Intervention Youth Mental Health Services: Evaluation Study
Source: J Med Internet Res. 2018 Sep 10;20(9):e259. doi: 10.2196/jmir.9966 (PMC6231849; doi:10.2196/jmir.9966)
Supplement: Multimedia Appendix 1 [file jmir_v20i9e259_app1.pdf]

DASHBOARD INFORMED INTERVIEW ITEMS

Assess the following items as per the individual's dashboard:

- Psychological distress
- Suicidal thoughts and behaviors
- Psychosis
    - Subthreshold symptoms
- Hypomania
    - Subthreshold symptoms
- Functioning
    - Use SOFAS as a reference
- Self-harm
- Tobacco use
- Alcohol use
- Social connectedness
- Depression
    - Subthreshold symptoms
- Anxiety
    - Subthreshold symptoms
    - Avoidance
- Physical health
- Sleep
    - Circadian disturbance
- Posttraumatic stress disorder
- Eating behaviors
- Other drug use
- History
    - Earlier onset neurodevelopmental or attentional disorders
    - Hospitalizations
    - Family history
