# Supplementary figures and images for: Using New and Innovative Technologies to Assess Clinical Stage in Early Intervention Youth Mental Health Services: Evaluation Study
Source: J Med Internet Res. 2018 Sep 10;20(9):e259. doi: 10.2196/jmir.9966 (PMC6231849; doi:10.2196/jmir.9966)

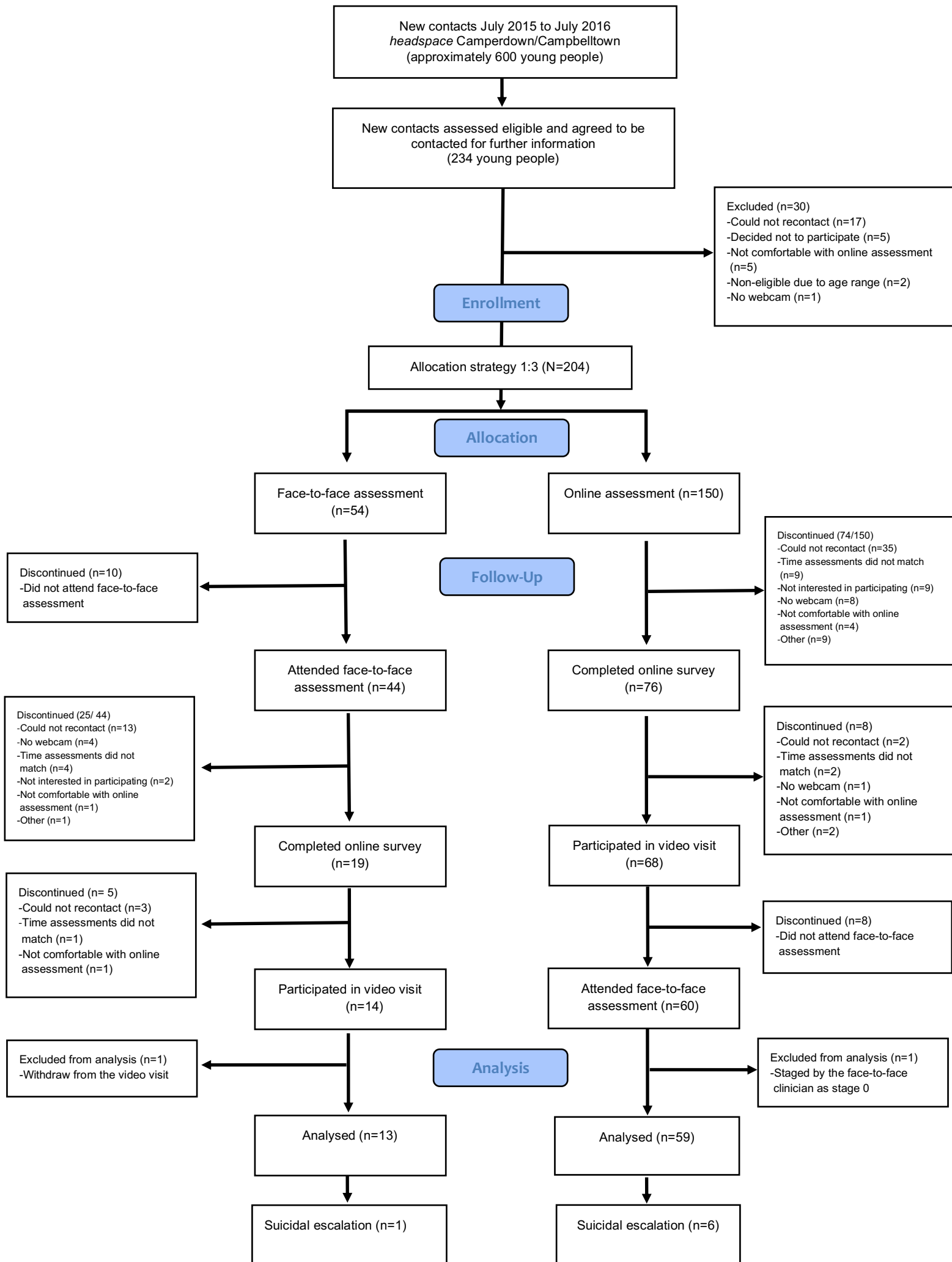

Supplement: Multimedia Appendix 2 [file jmir_v20i9e259_app2.pdf]
